# Students With High Metacognition Are Favourable Towards Individualism When Anxious

**DOI:** 10.3389/fpsyg.2022.910132

**Published:** 2022-05-18

**Authors:** Mauricio S. Barrientos, Pilar Valenzuela, Viviana Hojman, Gabriel Reyes

**Affiliations:** ^1^Cognitive Science Laboratory, Faculty of Psychology, Universidad del Desarrollo, Santiago, Chile; ^2^Faculty of Psychology, Universidad del Desarrollo, Santiago, Chile

**Keywords:** metacognition, problem-solving, social interdependence, anxiety, virtual classroom

## Abstract

Metacognitive ability has been described as an important predictor of several processes involved in learning, including problem-solving. Although this relationship is fairly documented, little is known about the mechanisms that could modulate it. Given its relationship with both constructs, we decided to evaluate the impact of self-knowledge on PS. In addition, we inspected whether emotional (self-reported anxiety) and interpersonal (attitudes towards social interdependence) variables could affect the relationship between metacognition and problem-solving. We tested a sample of 32 undergraduate students and used behavioural tasks and self-report questionnaires. Contrary to the literature, we found no significant relationship between metacognition and problem-solving performance, nor a significant moderating effect when including emotional and interpersonal variables in the model. In contrast, we observed a significant moderating model combining metacognition, self-reported anxiety and attitudes towards social interdependence. It was found that participants with high metacognition reported attitudes unfavourable towards interdependence when they felt high anxiety. These results suggest that already anxious individuals with high metacognition would prefer to work alone rather than with others, as a coping mechanism against further anxiety derived from cooperation. We hypothesise that in anxiogenic contexts, metacognition is used as a tool to compare possible threats with one’s own skills and act accordingly, in order to maximise one’s own performance. Further studies are needed to understand how metacognition works in contexts adverse to learning.

## Introduction

Metacognition refers to an individual’s ability to know their own mental states (Flavell, 1979). Every day we generate metacognitive outcomes by estimating several aspects of our behaviour, such as the time elapsed prior to making a decision (Corallo et al., 2008), the effort required to make it (Naccache et al., 2005) or the degree of confidence associated with it (Koriat et al., 1980). This phenomenon exhibits individuals’ conscious access to their own mental states (Proust, 2013), where these pieces of subjective information are critical to behavioural planning and control (Nelson and Narens, 1990). Now, metacognition has an impact on how we relate to others (Nichols and Stich, 2003; Shea et al., 2014) and in fields where said interactions are relevant, such as education (Norman et al., 2019). In particular, in educational sciences, it has been reported that individual metacognitive ability is one of the best predictors of learning and academic performance, even above other cognitive and motivational variables (Wang et al., 1990; Dent and Koenka, 2016; Ohtani and Hisasaka, 2018). Along the same lines, a positive relationship between the use of metacognitive skills and problem-solving performance has also been reported (Davidson and Sternberg, 1998; Bakar and Ismail, 2020).

Regarding the latter, problem-solving (hereinafter, PS) is a research topic that has widely captured attention in cognitive science and education (Jonassen and Hung, 2012). It has been attributed a fundamental role in learning processes (Anderson, 1993), and today, it is considered a relevant skill for the development of competencies in the 21st century (OECD, 2019). In cognitive science, PS constitutes an act involving the execution of a complex, multi-step sequence of goal-oriented processes, such as evaluating and planning, to arrive at an unknown solution (Bartley et al., 2018). Consistently, in education, PS has been defined as a capacity to engage in cognitive processes aimed at understanding and solving situations that do not have an obvious method of resolution (OECD, 2013). Although everything indicates that PS is a relevant construct to understand school achievement, there is no clarity regarding how it participates in the cognitive architecture of learning. One possibility suggested in literature is that cognitive, metacognitive and motivational processes are involved in SP (Mayer, 1998). The first refers to the set of processes involved in the processing, representation and resolution of the problem; the second, to the monitoring and control of cognitive processes; while the last one, to the emotional disposition of who solves the aforementioned problem. This model agrees with literature that suggests that the relationship between metacognition and SP occurs especially on complex problems that require a wide deployment of cognitive resources (i.e., non-insight problems), and not so on insight problems, in which the solution emerges spontaneously in consciousness (Metcalfe and Wiebe, 1987). In this way, metacognition would play an active and continuous role in the conscious administration of the cognitive resources used during the resolution of a problem (Stuyck et al., 2022). Given the importance that has recently been given to the study of metacognition and PS (English and Gainsburg, 2015; Perry et al., 2018; Azevedo, 2020), and its already mentioned connection, it becomes relevant to combine both phenomena in a single study that aims to clarify their relationship.

On the other hand, different studies have shown that the way in which metacognition is operationalised and evaluated is relevant to understand the impact it has on skills, such as academic performance, learning or SP itself (Dent and Koenka, 2016; Ohtani and Hisasaka, 2018; Bakar and Ismail, 2020). Precisely, these studies have shown that online measures, for example experimental tasks and think-aloud protocols, are better predictors of these variables than off-line measures, for example self-report questionnaires and interviews. This was early noted by Brown et al. (1983), who questioned the validity and reliability of off-line measurements arguing that they would assess beliefs regarding the skills, rather than the skills themselves. Due to this, in this article, we intend to study the relationship between metacognition and PS using instruments of both types. Similarly, literature in the area describes emotional and interpersonal processes that interact with both metacognition and SP and therefore could affect the reported relationship between both constructs. Regarding emotional processes, recent research has reported that these can alter the metacognitive capacity of individuals, specifically, high levels of stress and anxiety are negatively associated with the efficiency in monitoring one’s own mental states (Reyes et al., 2015, 2020; Barrientos et al., 2020; Culot et al., 2021). Similar results have been reported in the study of PS, where a negative influence of stress on PS performance has been evidenced (Alexander et al., 2007; Nair et al., 2020). Another possible modulator of this relationship are interpersonal variables, such as cooperation. In this regard, it has been reported that metacognitive monitoring training in cooperative learning contexts display better results than in competitive or individualistic learning contexts (Pesout and Nietfeld, 2021). This triad made up of cooperation, competition and individuality has its roots in the theory of social interdependence, widely used to guide instructional design towards cooperative environments and thus favour the development of competencies for the 21st century (Johnson and Johnson, 2014). In turn, it has also been suggested that collaborative social interactions would be beneficial for processes, such as decision-making and PS (Sills et al., 2016; Bang and Frith, 2017; Haataja et al., 2021).

Based on the above and given that, as far as we know, the literature in the area exhibits a lack of studies that focus on the emotional and interpersonal mechanisms that could affect the relationship between metacognition and SP, we designed a study that explores these relationships in undergraduate students. For this purpose, we used a set of self-report questionnaires and behavioural tasks. To assess the participants’ metacognition, we used both off-line and online measurements. For the former, we used a self-report questionnaire, the Metacognitive Awareness Inventory (Schraw and Dennison, 1994). For the latter, we implemented a 2-AFC computer task in which the participants had to indicate their confidence in their own decisions (Fleming et al., 2010). For PS, we designed a non-insight problem-solving task *ad hoc* to the disciplinary context of the participants. Finally, as emotional and interpersonal variables, we evaluated the self-reported feeling of anxiety (STAI-S; Spielberger et al., 1968), positive and negative affects (PANAS; Watson et al., 1988) and attitudes towards social interdependence in the classroom (Johnson and Norem-Hebeisen, 1979). Our hypothesis is that individuals’ metacognition will be a reliable predictor of PS, in line with what is reported in the literature. We also hypothesise that the aforementioned relationship will be modulated by anxiety, affects and attitudes towards social interdependence.

## Materials and Methods

### Participants

32 first-year undergraduate students (27 women) participated in the study. The average age of the participants was 19.97 years [SD = 2.02, *range* = (18–26)]. Although the initial sample consisted of 100 undergraduate students, only 32 answered all the questionnaires and correctly performed the online metacognition task. All participants had normal or corrected-to-normal vision. They received no direct compensation for their participation, although a prize (~ $150) was raffled among participants who completed the study. All participants gave written informed consent to participate in this study. The study was approved by the Comité de Ética Institucional en Investigación at Universidad del Desarrollo.

### Instruments and Procedure

#### Session 1

Participants were asked to respond to a perceptual task to evaluate their metacognitive efficiency and a series of self-report questionnaires to evaluate their metacognitive awareness, attitude towards social interdependence and state anxiety levels. Both the task and the questionnaires were coded using PsychoPy (Peirce et al., 2019) and uploaded to the Pavlovia webpage. Participants had 2 weeks to access the tasks, through a link to the webpage provided by a research assistant, on a computer or laptop in a quiet and dimly lit place.

##### Perceptual Confidence Task

**Figure 1:**
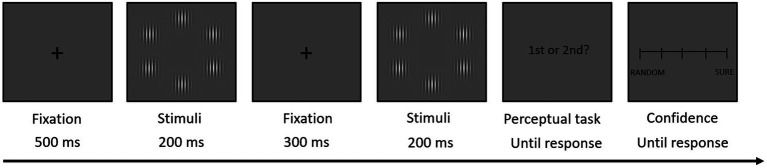
Structure of a single trial in the perceptual confidence task. Participants viewed each stimuli array during 200 ms and had to decide which had a different contrast Gabor patch. Immediately after their response, participants had to evaluate their confidence level in their own decision on a continuous scale from ‘at random’ to ‘secure’. The task comprised 50 training and 300 experimental trials.

Stimuli were arrays of six vertical Gabor patches on a grey background, presented on an imaginary circle at the centre of the screen. Participants were asked to perform the task in a dimly lit room and at a distance of 50 cm from the monitor. The task consisted in deciding in which of two arrays of Gabor patches—presented in a sequence—one Gabor patch with higher contrast was presented. After that, participants were asked to estimate their confidence in their decision (Figure 1). The experimental session comprised six blocks of 50 trials each with a pause between each block. The structure of each trial was the following: after a fixation cross (500 ms), participants were presented the two arrays for 200 ms each, separated by an interval of 300 ms. In one of the arrays, one random Gabor patch had a higher contrast. Participants had to decide when was presented that Gabor, by pressing the ‘Q’ (first array) or ‘W’ (second array) key on their keyboard. During the experiment, contrast varied on a trial-by-trial basis according to a 1-up 2-down staircase method with the aim of adjusting the individuals’ performance to 71% (Garcia-Pérez, 1998). After their response, participants were asked to give an estimate of their confidence about their decision on a scale from 1 (‘totally random’) to 5 (‘completely sure’).

##### Self-Report Questionnaires

*Metacognitive Awareness Inventory (MAI).* This scale assesses beliefs about one’s own cognitive monitoring and regulation (Schraw and Dennison, 1994). The version used in this study has 52 items, with Likert-type responses that range from 1 to 5 and was translated to Spanish and validated by Huertas et al. (2014), which showed a high internal reliability for the instrument (Cronbach’s 𝛼 = 0.94).

*Social Interdependence Scales.* These three scales evaluate the attitudes of students towards cooperation, competition and individualism in the classroom (Johnson and Norem-Hebeisen, 1979). The scales have 22 items and high internal reliability indices (Cronbach’s 𝛼 = 0.84, 85 and 0.88, respectively).

*State–Trait Anxiety Inventory, State Version (STAI-S).* This scale assesses state anxiety, that is the transitory experience of anxiety in the moment the participant answered the questionnaire (Spielberger et al., 1968). The Chilean version (Vera-Villarroel et al., 2007) has 20 items, with Likert-type responses that range from 1 to 4 and a high internal reliability (Cronbach’s 𝛼 = 0.92).

*Positive and Negative Affect Schedule (PANAS).* This scale assesses both positive and negative dimensions of affect (Watson et al., 1988). The Chilean version (Dufey and Fernández, 2012) has 20 items, with Likerttype responses that range from 1 to 5, and a high internal reliability for both scales (Cronbach’s 𝛼 = 0.86 and 0.88).

#### Session 2

About 2 weeks after they were asked to complete session 1, participants were instructed to perform a non-insight problem-solving task.

##### Problem-Solving Task

We developed a non-insight, open-ended problem-solving task related to a specific class from the student’s career. Two lecturers from said class helped us with the design of the task and allowed us to evaluate participants during class hours. The task begins with a description of a problematic situation, followed by four questions. Each one was aimed to assess a different process involved in problem-solving (OECD, 2013), namely, (a) exploring and understanding, (b) representing and formulating, (c) planning and executing and (d) monitoring and reflecting. The rubric described four performance levels for each question, which were scored from 1 to 4. The total score for this problem corresponds to the sum of the scores obtained in each question.

### Statistical Analysis

All analyses were performed using JASP v.0.13.1 (JASP Team, 2022) and R v.4.1 (R Core Team, 2021). Cronbach’s alpha showed an acceptable reliability level on all scales (all 𝛼 > 0.72). Two participants were excluded from analysis due to being outliers in several questionnaires at a time. In order to calculate metacognitive efficiency, we used the perceptual confidence task scores to calculate meta-d’/d’ for all participants (Fleming and Lau, 2014). This measure is an SDT approach and is conceptualised as the second-order (i.e., confidence) sensitivity relative to the first-order (i.e., perceptual task) sensitivity (Maniscalco and Lau, 2012). It represents an unbiased measure of an individual’s ability to monitor their own performance through the confidence in their decisions. We used Matt Craddock’s R port of Maniscalco and Lau’s Matlab functions^1^ and thank him for making these available to the community.

In order to obtain a robust measure of social interdependence, we calculated a composite score combining the three scales from the Social Interdependence Scales questionnaire. Given the theoretical valences of these three scales, we calculated social interdependence as the subtraction of the average of the z-scores of cooperation and competition minus the z-score of individualism. That way, positive values mean a favourable attitude towards interdependence, while negative values mean an attitude towards independence.

## Results

First, we analysed linear associations between metacognition and PS, attitudes towards social interdependence (Cooperation, Competition, Individualism and the composite measure), and emotional indexes (STAI and PANAS). We inspected how metacognition, operationalised as metacognitive efficiency and metacognitive awareness (MAI), predicts PS performance. Contrary to our expectations, results indicated no significant correlation between metacognitive efficiency and PS performance (*p* > 0.453). Same analyses repeated over metacognitive awareness reveals the same pattern: no significant association between both constructs (*p* > 0.959). Then, we inspected a possible association between metacognition and emotional indexes. We found that metacognitive efficiency showed a negative and moderate correlation with self-reported anxiety (*r* = −0.413, *p* < 0.05), meanwhile metacognitive awareness showed no relation with any of those indexes (all *p*s > 0.158). Finally, we investigated metacognition and social interdependence. Metacognitive efficiency showed no significant association with the composite measure of social interdependence or its subscales (all *p*s > 0.119), and metacognitive awareness only showed a marginal significant correlation with the cooperation subscale (*r* = 0.346, *p* = 0.061). These results are summarised in Supplementary Figure 1. Interestingly, we analysed and found no relation between both online (i.e., metacognitive efficiency) and off-line (i.e., MAI and its subscales) measures of metacognition (all *p*s > 0.137). However, further analysis using Bayes factors and the alternating conditional expectations algorithm (ACE; Breiman and Friedman, 1985) suggests that this may result from a lack of statistical power rather than a true null effect (all *BF_01_* < 1.85), particularly given the small sample size.

**Figure 2:**
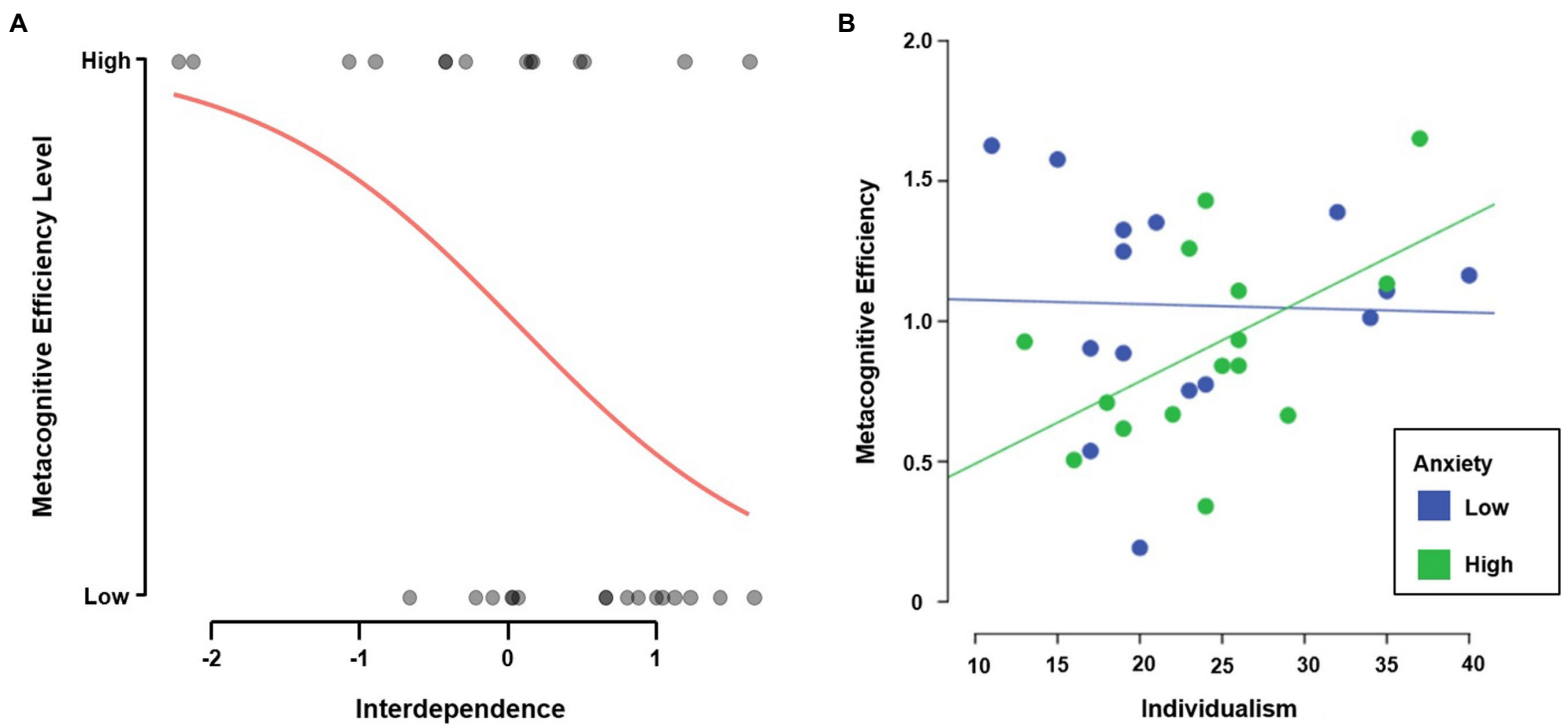
**(A)** Logistic regression of interdependence on metacognitive efficiency levels. **(B)** Regression of individualism on metacognitive efficiency by self-reported anxiety levels. *N* = 30.

In order to further inspect the relationship between metacognitive efficiency and the other variables studied, we factorised the metacognitive efficiency scores in two groups: a group with *low* metacognition (*n* = 16, *M* = 0.693, SD = 0.215) and a group with *high* metacognition (*n* = 14, *M* = 1.313, *SD* = 0.204). We used a score of 1.0 as the threshold to separate both groups, following Fleming and Lau (2014) who proposed it as a theoretically ideal value of metacognitive efficiency. Logistic regression was used to analyse if metacognitive efficiency levels were related to PS, interdependence and emotional indexes. We found that social interdependence predicted metacognitive efficiency levels (see Table 1). Indeed, the odds of having a high metacognition decreased almost three times for each 1-point increment in social interdependence (*OR* = 0.324, 95% CI [0.114, 0.920], *p* < 0.05). Next, and given its relationship to metacognition, self-reported anxiety was added as a predictor to this model in order to better explain the results. The resulting model was significantly better than the first one (*ΔΧ*^2^ = 6.157, *p* < 0.05, *R*^2^*_CandS_* = 0.337). It was found that, controlling by self-reported anxiety, the odds of having a high metacognition decreased almost four times for each 1-point increment in social interdependence (*OR* = 0.261, 95% CI [0.074, 0.923], *p* < 0.05). Likewise, controlled by social interdependence, the odds of having a high metacognition decreased 11% for each 1-point increment in self-reported anxiety (*OR* = 0.899, 95% CI [0.815, 0.991], *p* < 0.05). These results suggest that individuals with high metacognition tend to exhibit a poorer attitude towards social interdependence behaviours, such as cooperation or competition (see Figure 2A).

**Table 1:** Hierarchical logistic regression of metacognitive efficiency levels with interdependence and self-reported anxiety as predictors. *N* = 30.

Model	Variables	B	SE	Exp(B) with 95% CI	*R* ^2^
1	Interdependence	−1.126	0.532	0.324^*^ (0.114–0.920)	0.186^*^
					
2	Interdependence	−1.345	0.645	0.261^*^ (0.074–0.923)	0.337^*^
	Anxiety	−0.107	0.050	0.899^*^ (0.815–0.991)	

* *p* < 0.05: *N* = 30.

*R*^2^ calculated by Cox-Snell formula.

For simplicity, we investigated if a single subscale of social interdependence could better explain these results. We found that, although by itself it could not predict metacognitive efficiency levels (*p* = 0.057), Individualism was a significant predictor when controlled by self-reported anxiety. Specifically, the analyses showed that the odds of having a high metacognition increased 19% for each 1-point increment in individualism (*OR* = 1.187, 95% CI [1.008, 1.398], *p* < 0.05), if controlled by the anxiety scores. This finding reinforces what was previously reported and shows that the effect on metacognitive efficiency levels depends mainly on an increase in attitudes favourable to *independence*, rather than a decrease in attitudes favourable to *interdependence*. It also shows that self-reported attitudes towards individualism and self-reported anxiety are intertwined in their relation to metacognition.

Finally, to understand the relationship between these three variables, a three-stage hierarchical linear regression was conducted with metacognitive efficiency as the dependent variable. Individualism was entered at stage one, self-reported anxiety at stage two and their interaction at stage three. The analysis showed that individualism was not a significant predictor of metacognition by itself (*p* = 0.347). Incorporating self-reported anxiety to the model explained an additional 17.5% of variance [*ΔF*(1,27) = 5.97, *p* < 0.05]. Finally, the incorporation of the interaction between individualism and self-reported anxiety explained an additional 11.4% of variance [*ΔF*(1,26) = 4.36, *p* < 0.05]. When all predictors were included in the model, self-reported anxiety and its interaction with individualism were proved to be statistically significant predictors of metacognitive efficiency (both *p*s < 0.05). As seen in Table 2, the final model itself was also statistically significant and predicted a 32.1% of the variance of metacognitive efficiency [*F*(3,26) = 4.09, *p* < 0.05]. At last, we analysed the relationship between metacognitive efficiency and individualism after dividing self-reported anxiety by its median (Figure 2B). We found that the group with higher anxiety (*n* = 15, *M* = 50.67, SD = 7.59) showed a positive relation between metacognition and individualism (*r* = 0.529, *p* < 0.05), meanwhile the group with lower anxiety (*n* = 15, *M* = 32.4, SD = 6.07) showed no relation at all (*p* = 0.911). These results suggest that individuals with high metacognition tend to have attitudes favourable towards individualism, but only when they feel high anxiety.

**Table 2:** Hierarchical linear regression on metacognitive efficiency with individualism, self-reported anxiety and their interaction as predictors.

Model	Variables	*B*	SE	**β**	*R* ^2^	Δ*R*^2^
1	Individualism	0.009	0.010	0.178	0.032	–
						
2	Individualism	0.010	0.009	0.190	0.207^*^	0.175^*^
	Anxiety	−0.014	0.006	−0.419^*^		
						
3	Individualism	−0.05	0.03	−0.975	0.321^*^	0.114^*^
	Anxiety	−0.05	0.018	−1.524^*^		
	Interaction	0.002	0.001	1.664^*^		

* *p* < 0.05: Statistical significance. *N* = 30.

## Discussion

The main goal of this paper was to study the relation between PS performance and two different measures of metacognition. These two measures were metacognitive awareness, evaluated through the MAI (Schraw and Dennison, 1994), and metacognitive efficiency (Fleming and Lau, 2014). To better understand the relationship between both constructs, we also considered the attitude of the students towards social interdependence, self-reported anxiety and positive and negative affects.

The analyses showed no relationship between PS performance and metacognition. Although positive relations between both variables have been vastly observed and discussed (Davidson and Sternberg, 1998; Veenman, 2012; Azevedo, 2020), other studies have failed to show these results before (Jacobse and Harskamp, 2012). We hypothesised that this result could be caused by two main reasons. On the one hand, different measurements of metacognition have shown to not relate in the same way to PS. For example, Jacobse and Harskamp (2012) investigated how three measurements of metacognitive ability (two online and one off-line) predicted PS performance. They found that, even though the two online measures—a think-aloud protocol and a novel instrument—were strongly related to PS, the off-line measure—a self-report questionnaire—was not related to it. Similarly, recent meta-analyses have shown that off-line measures of metacognition are worse predictors of learning and academic performance than online measures (Dent and Koenka, 2016; Ohtani and Hisasaka, 2018). Those results point out that a subjective measurement of metacognition—such as the MAI—may not predict concrete skills, such as PS performance. With respect to the online measure, although we had one, metacognitive efficiency has been scarcely used in educational studies and its relationship to other measures has not been studied thoroughly (Fleur et al., 2021). Furthermore, in comparison to other online measures, metacognitive efficiency has some qualities that make it different, namely, it lacks the biases that judgements of confidence or learning have, its independent from first-order performance, and its relationship to consciousness is not well known (Rahnev et al., 2021). A possibility is that metacognitive efficiency—evaluated by a perceptual task—represents a primary self-regulatory mechanism without conscious access to cognitive processes (*cf.* Nisbett and Wilson, 1977) and, as that, does not have an impact in the way in which individuals modulate more complex processes, such as PS and learning. If that is true, it remains to be seen if evaluating metacognitive efficiency *via* more cognitively complex tasks, such as a memory task (Fleming et al., 2014), brings better results. Finding this relation would mean that specific aspects of the memory metacognitive efficiency—probably shared with other online measures of metacognition, but not with perceptual metacognitive efficiency (Rouault et al., 2018)—are related to PS performance.

Interestingly, we found a negative relationship between metacognitive efficiency and self-reported anxiety. This result comes to reinforce previous findings (Reyes et al., 2015, 2020; Barrientos et al., 2020; Culot et al., 2021) and give relevance to the inclusion of emotional variables in similar studies. Indeed, the COVID-19 pandemic has had an impact in the anxiety that students report (Yang et al., 2021) and in the coping mechanisms they use (Babicka-Wirkus et al., 2021), which lead us to the main finding of this study. We found a negative relationship between metacognitive efficiency and self-reported attitudes towards social interdependence in the classroom. Further analyses evidenced that this relationship was mostly explained by individualism which showed a positive relationship to metacognitive efficiency. In other words, participants who are more skilled in monitoring their own mental states declare to be more favourable towards individualistic behaviours. Furthermore, the data shows that this relationship is more common for students that report feeling high anxiety levels. While the bulk of literature in metacognition and social interdependence tend to focus on the positive aspects of the interrelationship between cooperation and metacognition (Frith, 2012; Grau et al., 2018; Smith and Mancy, 2018), our findings give hints that point in the opposite direction. We hypothesise that, in an already anxiogenic context, individuals with high metacognition would prefer to work alone rather than with others, as a coping mechanism against further anxiety derived from collaborative work. Speculatively speaking, individuals with high metacognition could be better at reading their environment and own capabilities and adapting to changes in any of them, in order to maximise their performance. It remains to be seen if this is true, and the effect is not explained by personality factors, such as behavioural inhibition that could lead participants to retreat from social interactions in anxiogenic contexts.

Sociocultural practices, such as collaboration or cooperative learning, are developed in a setting of community activity or conditions, and it is these conditions that modulate how this collaboration develops (Rogoff, 1998; Mejía-Arauz et al., 2018; Hedegaard, 2019). In other words, the relationship that people have with collaboration and the way in which it is performed is dialectically defined by the social or interactional context in which this activity is carried out. In the Chilean case, the educational scenario has been described as an individualistic scenario, where the educational policy’s logic is framed in accountability and individual incentives (López et al., 2018), which also reflects a tendency towards classroom methodologies that are more individual and teacher-centred than collective and focused on interaction between students (Preiss, 2011; Martinic et al., 2013). It is possible that students feel comfortable in individual learning spaces, while in collective contexts they feel in an unknown territory where they have fewer tools available. Thus, in an anxiogenic time, such as the context of a pandemic, with computer-mediated interaction and, with peers who do not know each other face to face, it could be expected that students prefer not to participate in group activities, even more so students with a high metacognitive efficiency, that is, students that know what they know and what they do not.

Nonetheless, our study had limitations, namely, the low sample size and the instruments’ selection. The low sample size could lead us to not find relations between variables due to their low effect and/or the low statistical power of our analyses (i.e., type-II errors). Indeed, post-hoc analyses and a review of studies with similar variables suggest a sample size at least twice as large (Desender and Sasanguie, 2022; Taouki et al., 2022). With respect to the instruments’ selection, our study relied too heavily on self-report questionnaires. Those have been criticised for being inherently biased and representing beliefs rather than the processes themselves (Craig et al., 2020). Studies of this type would benefit from objective measures of emotional distress and interdependence in participants. In line with this, future research should aim at replicating this study in classrooms to evaluate if the attitudes towards interdependence translate into social behaviours coherent with them. It would be interesting to see if these attitudes affect the quantity and quality of interactions in the classroom, and how they relate to the metacognitive ability of the students. In the same line, an experimental replication manipulating the stress level that participants feel could help us understand if individuals with high metacognition are better suited to adapt to changes in their environment, being collaborative in favourable contexts and individualistic in adverse ones. Finally, further focus should be put on studying how metacognitive efficiency relates to (a) problem-solving skills and (b) other measures of metacognition. Integrating what different academic fields know about metacognition should be a priority, in order to better understand, evaluate and train this ability in various settings. As far as we know, there is a lack of literature that seeks to explain what we know about metacognition in educational settings. Even though the positive influence of metacognition in aspects, such as learning, academic performance or PS, is widely known, the mechanisms that explain these relationships are not well documented. In the same line, despite being extensively used and studied in cognitive sciences and neuroscience, metacognitive efficiency, and its gold-standard measure: meta-d’/d’, have been rarely used in education. We think that its use and study in educational studies should be encouraged as a way to build conceptual and methodological ‘bridges’ between both fields.

## Data Availability Statement

The datasets presented in this study can be found in online repositories. The names of the repository/repositories and accession number(s) can be found at: https://osf.io/ajb6c/.

## Ethics Statement

The studies involving human participants were reviewed and approved by Comité de Ética Institucional en Investigación, Universidad del Desarrollo. The patients/participants provided their written informed consent to participate in this study.

## Author Contributions

MB and PV collected the data. MB and GR analysed the data. All authors drafted the article, contributed to the study concept and design, and approved its final version for submission.

## Funding

This work was supported by a research grant from the Centro de Innovación Docente (CID) at Universidad del Desarrollo to MR and VH and by ANID Becas/Doctorado Nacional 21211819 to MB.

## Conflict of Interest

The authors declare that the research was conducted in the absence of any commercial or financial relationships that could be construed as a potential conflict of interest.

## Publisher’s Note

All claims expressed in this article are solely those of the authors and do not necessarily represent those of their affiliated organizations, or those of the publisher, the editors and the reviewers. Any product that may be evaluated in this article, or claim that may be made by its manufacturer, is not guaranteed or endorsed by the publisher.

## Supplementary Material

The Supplementary Material for this article can be found online at: https://www.frontiersin.org/articles/10.3389/fpsyg. 2022.910132/full#supplementary-material

## Footnotes

^1^ https://github.com/craddm/metaSDT
