# Supplementary material for: Students With High Metacognition Are Favourable Towards Individualism When Anxious
Source: Front Psychol. 2022 May 18;13:910132. doi: 10.3389/fpsyg.2022.910132 (PMC9158479; doi:10.3389/fpsyg.2022.910132)
Supplement: Supplementary file 1 [file Table_1.DOCX]

Supplementary Material

**Supplementary Figure 1**


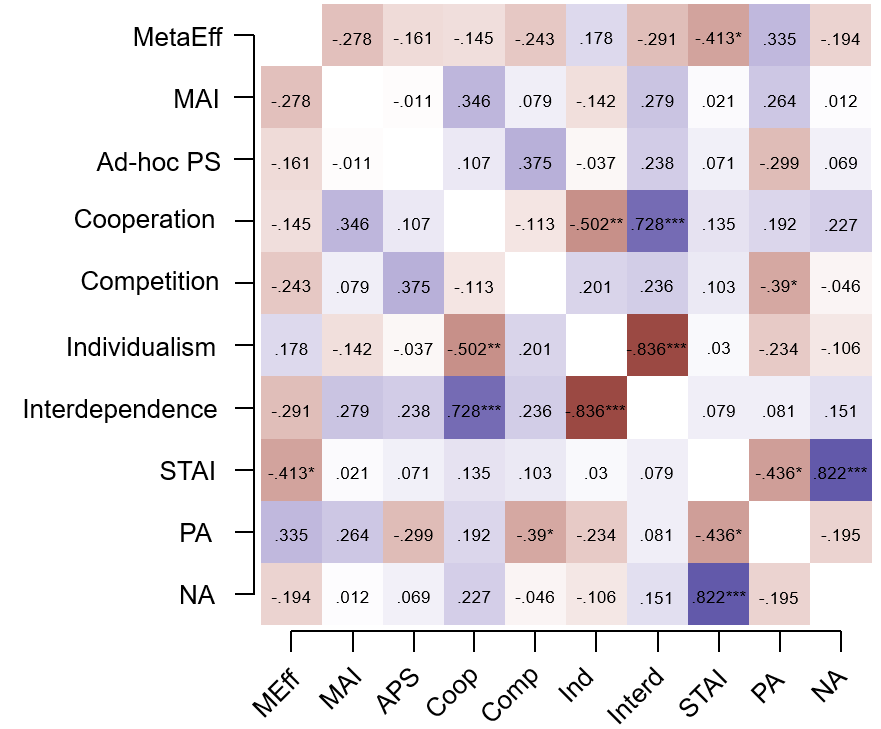


**Supplementary Figure 1.** Heatmap of linear regression coefficients. MetaEff and MAI represent both measures of metacognitive ability. Ad-hoc PS represents the total score of the problem-solving task. Cooperation, Competition and Individualism are the subscales of the Social Interdependence Scale, while Interdependence equals the composite measure obtained from them. Finally, STAI, PA and NA are three measures of emotional indexes: self-reported anxiety, positive affect and negative affect, respectively. *N* = 30.
